# A Neurophysiological and Behavioral Assessment of Interventions Targeting Attention Bias and Sense of Control in Binge Drinking

**DOI:** 10.3389/fnhum.2018.00538

**Published:** 2019-01-11

**Authors:** Jessica E. Langbridge, Richard D. Jones, Juan J. Canales

**Affiliations:** ^1^Department of Psychology, University of Canterbury, Christchurch, New Zealand; ^2^New Zealand Brain Research Institute, Christchurch, New Zealand; ^3^Division of Psychology, College of Health and Medicine, University of Tasmania, Hobart, TAS, Australia

**Keywords:** attention bias, attention bias modification, binge drinking, alcohol drinking, ERP, sense of control

## Abstract

Attention bias modification (ABM) can decrease the selective visual attention paid to alcohol-related cues but has not been found to reliably reduce alcohol craving. Here, a cognitive intervention to decrease craving by increasing sense of control (Shamloo and Cox, 2014) was used as a complement. We investigated the effects of two such interventions administered singly or in combination. Participants were 41 binge drinkers (BDs) and 10 non-binge drinkers (NBDs). BDs received either ABM, sense of control training, both interventions, or no intervention, and were compared with NBDs who received no intervention. Groups were assessed on alcohol attention bias change including both reaction times and cue-elicited ERPs (visual dot-probe task), alcohol craving change, and alcohol consumption. BDs exhibited higher attention bias scores than NBDs. ABM had no effect on BDs’ behavioral or electrophysiological markers of attention bias. Sense of control training did not increase personal sense of control but protected against decreased task accuracy and against increased craving. BDs receiving the combined intervention consumed less alcohol in a bogus taste test than participants receiving no intervention. Taken together, the results suggest that ABM procedure may reduce alcohol consumption if combined with sense of control training.

## Introduction

Recurrent binge drinking is a hazardous behavior (Babor et al., 2010) that attracts social, media, research, and policy concern (Measham, 2008). Sustained binge drinking may lead to functional deficits that mirror those seen in dependent drinkers, such as impairments in working memory and verbal encoding (Petit et al., 2014). A deficit particularly relevant to the maintenance of a binge drinking pattern, and potential escalation to compulsive use, is attention bias for alcohol-related cues. According to the incentive-sensitisation theory, attention bias is a product of, and a contributing factor to, excessive alcohol use (Robinson and Berridge, 1993). Attention bias has a mutually excitatory relationship with craving (Field et al., 2009). Thus, reducing attention bias has attracted scientific attention as an intervention target for dependent and at-risk groups. The attention bias modification (ABM) method typically uses visuospatial cueing paradigms and was originally used to identify attention biases for threatening stimuli which characterize emotional disorders such as anxiety (MacLeod et al., 1986). Interestingly, some effects of ABM on retraining appetitive attention biases have also been reported (Beard et al., 2012). Moreover, ABM has been applied to drinkers with varying success (Townshend and Duka, 2001; Field and Eastwood, 2005; Field et al., 2007; Schoenmakers et al., 2007, 2010). However, despite reports of promising effects on neurophysiological measures, such as cue-elicited event related potentials (ERPs), there is limited evidence that ABM can significantly reduce subjective experiences such as craving (Beard et al., 2012; Mogoaşe et al., 2014).

A possible complement to ABM is an intervention to decrease craving by increasing sense of control, as reported with social and moderate drinkers. Shamloo and collaborators designed a novel manipulation to increase sense of control (dissertation, cited in Fadardi et al., 2011). Problem-solving-type tasks were delivered with additional instructions to encourage feelings of success. Social drinkers receiving the sense of control intervention were given choices, a time limit, information enhancement (i.e., how to go about problem-solving and achieving goals), and immediate and contingent reinforcement. These participants were more accurate on the tasks and, importantly, reported weaker urges to drink and showed less alcohol-related attention bias. This was replicated in a later study of moderate drinkers (Shamloo and Cox, 2014), where the manipulation increased sense of control, improved task accuracy, and decreased alcohol attention bias. This intervention emphasizes participants’ competence and contingencies, to show the environment as responsive (Skinner, 1996; McKean Skaff, 2007) and, if effectively combined with ABM training, it could compensate for ABM’s limitations. Consistent with the dual process conception of addiction (Stacy and Wiers, 2010), this combined intervention may be more effective by addressing both the automatic processes of attention bias (through ABM) and more controlled, top-down processes (through sense of control manipulation).

The present study aimed to investigate whether a combined intervention to increase sense of control and decrease alcohol attention bias would be more effective than either intervention alone in reducing alcohol craving, alcohol attention bias, and alcohol consumption. We investigated in a population of at-risk binge drinkers (BDs) the effectiveness of ABM training, sense of control treatment, and their combination, on a variety of neurophysiological and subjective measures. We hypothesized that BDs would show greater alcohol attention bias than non-binge drinkers (NBDs) (faster reactions and greater P1 and N1 amplitudes to alcohol-probes), and that ABM would decrease attention bias, as previously shown (Townshend and Duka, 2001; Field and Eastwood, 2005; Field et al., 2007; Schoenmakers et al., 2007, 2010). We further predicted that the sense of control intervention would increase personal and task-specific sense of control, improve accuracy on tasks used to deliver the interventions, and decrease craving. Lastly, we predicted that in a post-test challenge of alcohol consumption, participants receiving both interventions would drink less alcohol than participants receiving one intervention only, and less still than untrained participants.

## Materials and Methods

### Participants

Binge drinkers (BD; *n* = 41) and non-binge drinkers (NBD; *n* = 10) were compared on baseline variables and assigned to one of five experimental groups (Table 1). BDs were assigned to receive sense of control training only, attention bias training only, both trainings, or neither in a pseudorandom fashion: participants were assigned on joining the study, actively balancing groups for Alcohol Use Disorders Identification Test (AUDIT) scores [*F*(3,37) = 0.27, *p* = 0.846] and binge scores [*F*(3,37) = 0.63; *p* = 0.602]. NBDs completed tasks with neither training. The five experimental groups were matched for ratio of males to females, and on age (Mann-Whitney *U* = 203; *p* = 0.962). Table 2 gives descriptive statistics. The 51 participants aged 18–50 were recruited through posters and online advertisements in Christchurch, New Zealand, after screening in an online survey. All had AUDIT scores below 20 and reported no current psychiatric or regular recreational drug use, and no family history of alcoholism. All participants were given an information sheet prior to enrolling into the study and signed a written consent form. A debriefing form was also made available to all participants on completion of the study. Ethical approval was received from the University of Canterbury Human Ethics Committee.

**Table 1:** Experimental groups.

Experimental group	*n*	Drinking group	Sense of control intervention	Attention intervention	
1	10	BD	Training	Training	Combined intervention
2	10	BD	No training	Training	Attention training only
3	10	BD	Training	No training	Sense of control training only
4	11	BD	No training	No training	Untrained binge drinkers
5	10	Non-BD	No training	No training	Untrained non-binge drinkers


**Table 2:** Descriptive statistics for demographic and drinking variables.

Group	*n*	Age			AUDIT scores		Binge scores		Sex ratio (F:M)	Smokers
		*Mdn*	Range	Interquartile range	*M*	*SD*	*M*	*SD*		
1	10	21.5	20–25	1	10.70	4.42	41.04	17.46	6:4	0
2	10	21.0	18–42	5	12.30	4.17	40.60	7.03	6:4	2
3	10	21.5	18–25	6	11.50	4.30	38.36	9.01	6:4	1
4	11	20.0	18–48	4	11.27	3.20	34.82	10.71	7:4	0
BDs	41	21.0	18–48	4	11.44	3.92	38.61	11.54	25:20	3
Non-BDs	10	22.0	18–25	5	2.10	2.08	6.54	5.02	6:4	0
Total	51	22.0		4	9.61	5.20	32.32	16.62	31:24	3


#### Apparatus and Stimuli

Stimuli were prepared and presented with E-Prime (Professional suite; run-time version 2.0.10.353; Psychology Software Tools, Inc.) and displayed on a Philips 22-inch monitor (resolution 1860 × 1050) approximately 60 cm from participants. Event triggers were coded into the ABM task in E-Prime and sent via an Input/Output port to a Neuroscan EEG/EP system with a 64-channel SynAmps II headbox (Compumedics, Abbotsford, Australia) which recorded the EEG data via SCAN version 4.4.

#### ERP Recording

EEG data were recorded using an electrode cap arranged in the international 10–20 system (64-channel Quik-Caps; Compumedics Neuromedical Supplies, Abbotsford, Australia). Offline data processing was performed using MatLab (Version 2014b) with the EEGLab and ERPLab plug-ins. Data were filtered online at 1.0–40 Hz with a sampling rate of 1000 Hz and referenced online to a central midline electrode. A bipolar VEOG electrode monitored blinks and vertical eye movements. Final sites for analyses included Pz, Fz, a parietal cluster (averaging sites P5, P3, P1, Pz, P2, P4, and P6), and a frontal cluster (averaging F5, F3, F1, Fz, F2, F4, and F6). The median impedance was 15 kΩ (range 7–73 kΩ).

### Sense of Control Intervention

Two simple cognitive tasks, anagrams and concept identification cards (CIC), were used as vehicles for delivering the sense of control intervention elements, as in Shamloo and Cox (2014). Participants receiving the intervention (Groups 1 and 3) completed the tasks with additional instructions: these participants (a) chose the task order, (b) set time goals, (c) received information about the task nature (i.e., that tasks were skills-based and performance could be improved; hints about strategy), and (d) received immediate positive reinforcement. These intervention elements can be understood to highlight participants’ competence and contingencies, showing them as able to affect change, and showing their environment to be responsive to those changes, as is important to sense of control (Skinner, 1996; McKean Skaff, 2007). For example, intervention participants set their own time goals for each set of five anagrams or five concept cards and, being told that people can show improvement on these tasks, would often set increasingly challenging personal goals. Untrained groups (2, 4, and 5) completed the tasks without further instructions.

Anagrams were generated as in Shamloo and Cox (2014). Words were extracted from written and spoken English in the British National Corpus (Leech et al., 2001) in three categories: easy (frequency of 40–50 per million), moderate (20–40) and difficult (10–20). Five blocks of five words without repeated letters were selected and shuffled into letter-strings using a random shuffle order.

Replicating Shamloo and Cox (2014), CIC each depicted a shape varying in five dimensions (e.g., blue or orange color, circle or square shape). In five blocks of five trials, participants were tasked with identifying the dimension common to a pair of cards presented simultaneously (e.g., both striped pattern) by pressing a corresponding key. Card dimensions came from Hiroto and Seligman (Hiroto and Seligman, 1975). Cards were created for computer presentation on 300 × 300 px canvases with 75 × 75 px shapes.

### Attention Bias Intervention

**FIGURE 1:**
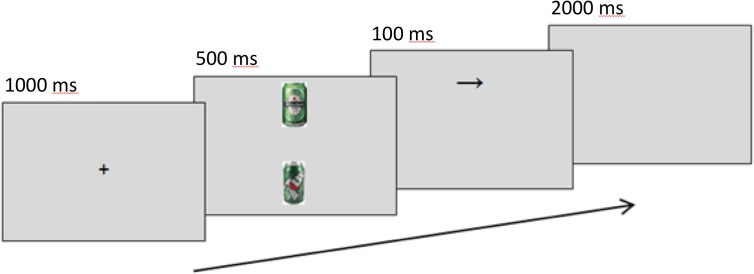
Sequence of events in a modified dot-probe trial for assessing alcohol attention bias. The example shows an alcohol trial.

A modified dot-probe task measured and manipulated attention bias. Trials present a 1000-ms centralized fixation cross, followed by a screen showing one alcohol-related image and one neutral image. Alcohol images were selected to include easily recognizable, locally available brands. Neutral matches for alcohol-related images were sourced using Google’s image search algorithms and “visually similar” search function with a category keyword and refined by eye for similar colors and composition. Irfan view was used to crop and resize images to a standardized height of 10 cm (378 px), and to edit images for brightness, contrast, and other color corrections where necessary. Alcohol-neutral image pairs were shown simultaneously for 500 ms in top-bottom formation. A 100-ms arrow probe (↑ or →) replaces one image, and participants are instructed to identify its orientation with a corresponding keyboard arrow key. This procedure is similar to Eldar and Bar-Haim’s (2010) method with (: or ^..^) as probes. Trials in which the probe replaced the alcohol-related cues are referred to as “alcohol-probes,” and trials where the probe replaced the neutral cue as “neutral-probes.” Attention bias scores were calculated as the difference in reaction times between the two conditions (RT_NeutralProbe_ – RT_AlcoholProbe_), where greater scores represent a greater focus on alcohol cues. Only correct responses 200–2000 ms after probe presentation were included in analyses. Figure 1 shows the task. Amplitudes of the P1 and N1 components at picture offset/probe onset, occurring around 80–130 ms and 140–200 ms, respectively, were used as markers of initial attention orienting, as they are greater when stimuli appear in attended locations rather than unattended locations (Hillyard and Anllo-Vento, 1998).

In pre- and post-tests, probes replaced image conditions equally in pre-test, post-test, and untrained blocks.

Participants receiving attention bias training were exposed to a manipulated arrow probe between the pre-test and post-test blocks of the ABM task: probes replaced the neutral image 80% of the time, compared to 50% as for the untrained groups.

After a 10-trial practice with neutral-neutral image pairs, participants completed seven blocks of 120 alcohol-neutral trials each: a pre-test, five blocks of training or no training, and a post-test. Blocks used alcohol-neutral image pairs (40 pairs total). Arrow probes replaced alcohol images on 50% of trials pre-test, post-test, and the five untrained blocks. In the five training blocks (ABM), probes replaced neutral images on 80% of trials. In a contingency test, participants who were aware of this manipulation when asked were excluded from analyses (*n* = 1). In pre- and post-tests for all participants, event triggers were emitted simultaneously with probe presentation, distinguishing between alcohol-replacing and neutral-replacing conditions. After offline EEG processing, ERPs were created with an average of 57 trials per condition per test per participant. Grand averages were weighted based on the number of trials.

### Measurements

#### Baseline Drinking Behaviors

A Binge Score, derived from the Alcohol Use Questionnaire (AUQ; Mehrabian and Russell, 1978), defined drinking groups as described by Townshend and Duka (2002): BDs scored 24 or above, and NBDs scored 16 or below. The AUQ operationalises binge drinking with a weighted sum of speed of drinking, frequency of drinking, and percentage of times that drinking leads to drunkenness. The AUQ correlates well with diary measures of consumption (Townshend and Duka, 2002). Its strength is its focus on drinking patterns rather than simple consumption,; Herring et al., 2008. as does the AUDIT-C (Bush et al., 1998). The Alcohol Use Disorders Inventory Test (Saunders et al., 1993) measured risk of alcohol-related harm and screened out individuals with possible dependence (i.e., those with scores of 20 or above; Babor et al., 2001).

#### Sense of Control

Two summary measures were developed to measure within-subjects change based on the Shapiro Control Inventory’s general domain sense of control and desire for control subscales (Shapiro, 1994). This follows Shamloo and Cox’s (2014) development of a task-specific control inventory. The five items selected were tested for reliability against SCI data from a pilot group (*n* = 12; 3 males and 9 females; mean age 23.36, range 18–29). The resultant measure was applied to personal sense of control (Summary-SCI) and to task-specific control (TSSCI; referring to the tasks used in each intervention).

#### Alcohol Craving

The 5-item Penn Alcohol Craving Scale (Flannery et al., 1999) assessed past-week craving. To measure change, a single-item time-locked craving question (TLC) asked how much participants currently craved an alcoholic drink on a scale of 0 (*not at all*) to 10 (*extremely*). Other ABM studies have done, similarly (Field and Eastwood, 2005).

#### Ad-Libitum Alcohol Consumption

In a “taste test” (Jones et al., 2016), participants filled out a bogus taste survey after sampling from 200 ml of an alcoholic beverage (Lion Brown draft beer, 4.0% alcohol; Lion) and from 200 ml of a non-alcoholic beverage (Just Juice orange fruit drink; Frucor Beverages Ltd.). Survey responses were discarded and the experimenter measured consumption after the session. Participation was voluntary (33 BDs and 4 NBDs). Two BDs identified the test’s purpose and were excluded from this analysis.

### Procedure

In afternoon testing sessions, lasting approximately 2 h, the researcher explained the session order, from the electrode cap fitting, to the computer-based personality-type questionnaires, then two problem-solving tasks (the sense of control tasks) and one longer visual task (the attention bias task). Informed consent was obtained before continuing. An experimental script guided the researcher’s instructions.

**FIGURE 2:**
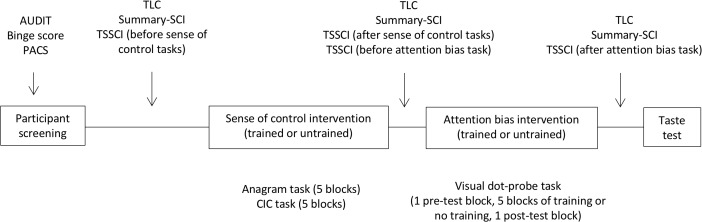
Experimental procedure. The screening survey captured drinking variables with the AUDIT scale of hazardous drinking and the binge score. Craving was measured at baseline with the Penn Alcohol Craving Scale (PACS), and as a repeated measure with the time-locked measure (TLC). Sense of control was measured at baseline with the Shapiro Control Inventory (SCI). Repeated measured of sense of control focused on personal sense of control (SummarySCI), and task-specific sense of control (TSSCI).

Penn alcohol craving scale (PACS) was administered once at baseline; TLC and Summary-SCI before, between and after the two interventions; and TSSCI before and after each intervention. The ad-libitum test was the final measure before debriefing. Figure 2 shows the experimental procedure.

### Data Analysis

A power analysis (significance set at 0.05, power of 80%) was conducted to calculate the appropriate sample size required (Cohen, 1988). Calculations were based on RTs and P3 measures from previous studies (Bartholow et al., 2010; Petit et al., 2012, 2013), yielding an estimated minimum sample size of *n* = 45 (including all five groups).

An independent *t*-test compared drinking groups on baseline attention bias. One-way ANOVAs compared experimental groups on baseline craving and task performance. Repeated-measures mixed ANOVAs assessed changes in craving, personal and task-specific sense of control, task accuracy and speed, and attention bias. Where variables were non-normally distributed, based on skewness or kurtosis values greater than 1.96 (Kim, 2013), equivalent non-parametric analyses were used. This concerned craving and task performance data (Kruskall-Wallis, Friedman, and Wilcoxon tests). *Post hoc* analyses of differences used Newman-Keuls tests. Effect sizes are given as Cohen’s *d* for parametric data, partial η^2^ ($\eta_{p}^{2}$) for interaction terms, and *r* for non-parametric data (Rosenthal et al., 2000; Rosenthal and DiMatteo, 2001).

#### ERPs

Offline data processing was performed using MatLab’s EEGLab and ERPLab plug-ins (version 2014b). EEG data were filtered at 1–30 Hz (48 dB/oct) and re-referenced to an average mastoid reference. All-channel noise was removed with the FASTER method (Nolan et al., 2010), and ICA was used to identify and remove ocular artifacts. Final EEG data had artifacts removed and noisy epochs rejected.

Analyses used average ERPs smoothed with a 50 ms moving window average to make the dominant peaks more perceptible (Delorme et al., 2015). P1 and N1 amplitudes were, respectively, defined as the most positive and negative peaks between 100 and 200 ms after probe presentation. Values falling before 100 ms were identified manually. Baseline comparisons used a 2 × 2 (Drinking group × Probe) ANOVA, and 5 × 2 × 2 (Experimental group × Probe × Time) ANOVAs examined change over time. The factor Time refers to assessments at two points: before the interventions were introduced, and after both were completed. Data from one non-binge drinker was excluded for unacceptable noise. Final analyses concerned 50 participants (41 BDs and 9 controls).

## Results

### Baseline Attention Bias

#### Behavioral

Binge drinkers (*M* = 4.24, *SD* = 14.07) showed higher alcohol attention bias scores than NBDs (*M* = -4.32, *SD* = 15.79), *t*(49) = 1.68, *p* = 0.049, *d* = 0.57.

#### ERPs

**FIGURE 3:**
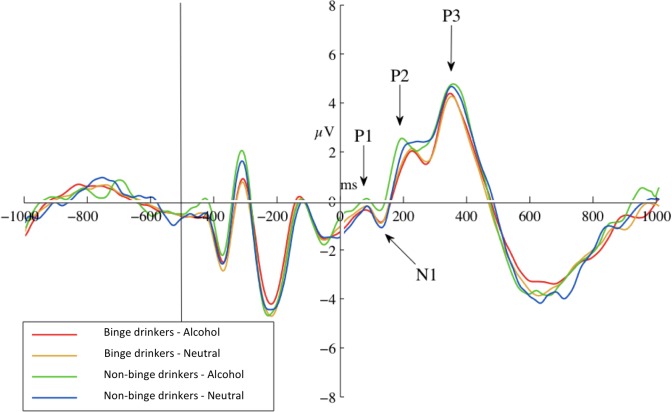
Pre-test ERPs at site F-Cluster. ERPs are averaged around probes (appearing at 0 ms and separated by probe condition: probes replacing alcohol images or probes replacing neutral images). Epoch length (–1000 to 1000 ms) covers true baseline (–1000 to 500 ms), picture pair presentation (–500 ms), and probe presentation (0 ms) and response.

The 2 × 2 (Probe-Group) ANOVA yielded no significant main or interaction effects for P1 at any site, or for N1 at parietal sites. Frontally, there was a marginal trend for greater N1 amplitudes in response to neutral-probes (probes replacing neutral cues) than alcohol-probes (probes replacing alcohol cues), at Fz, *F*(1) = 3.76*, p* = 0.058, *d* = 0.56, and the F-Cluster, *F*(1) = 4.21*, p* = 0.046, *d* = 0.59. Descriptive statistics are given in Table 3. A graph of F-Cluster at pre-test is shown in Figure 3.

**Table 3:** Baseline N1 amplitudes in frontal sites of analysis by drinking group.

		Pre-test				Post-test			
		Alcohol		Neutral		Alcohol		Neutral	
			
Site	Group	*M*	*SD*	*M*	*SD*	*M*	*SD*	*M*	*SD*
Fz	BDs	-2.00	2.61	-1.59	1.73	-2.33	2.37	-2.11	1.95
	Non-BDs	-1.59	1.73	-2.47	1.18	-1.59	2.22	-1.38	2.40
F-Cluster	BDs	-1.92	2.27	-2.17	2.16	-1.87	1.95	-1.72	2.04
	Non-BDs	-1.45	1.70	-2.22	1.07	-1.55	2.04	-1.36	2.32


The N1 probe-group interactions were not significant at either Fz, *F*(1) = 1.40, *p* = 0.243, $\eta_{p}^{2}$ = 0.028, or the F-Cluster, *F*(1) = 1.35, *p* = 0.252, $\eta_{p}^{2}$ = 0.027.

### Effects of ABM on Alcohol Attention Bias

#### Behavioral

The 2 × 2 (Time × Experimental Group) ANOVA revealed a main effect of Time, *F*(1) = 3.81, *p* = 0.029, *d* = 0.58: attention bias scores decreased from pre-test (*M* = 2.56, *SD* = 14.66) to post-test (*M* = -3.66, *SD* = 18.99) for all participants. Neither the Time × Experimental Group interaction, *F*(1) = 1.40, *p* = 0.125, $\eta_{p}^{2}$ = 0.109, nor the experimental group, *F*(4) = 0.05, *p* = 0.498, *d* = 0.13, had a significant effect.

#### ERPs

There was no three-way probe-time-experimental group interaction at any site for either the P1 or the N1 components, indicating no decrease in amplitudes for trained groups over time.

### Sense of Control Intervention on Sense of Control

The 3 × 5 (Time × Experimental Group) ANOVA of personal sense of control showed scores did not change significantly over time, *F*(5.801) = 0.33, *p* = 0.327, *d* = 0.31. The Time × Group interaction was not significant, *F*(5.801) = 0.86, *p* = 0.530, $\eta_{p}^{2}$ = 0.069, and the between-subjects factor of experimental group had no significant effect, *F*(4) = 0.87, *p* = 0.491, *d* = 0.55.

The 4 × 5 (Time × Experimental Group) ANOVA of task-specific sense of control showed a marginal effect of time, *F*(2.78) = 2.77, *p* = 0.044, *d* = 0.49. Scores were higher before the sense of control intervention tasks (*M* = 27.04, *SD* = 3.82) than after (*M* = 25.35, *SD* = 4.19), as identified by Newman-Keuls *post hoc* tests (*p* = 0.001). The Time × Group interaction was not significant, *F*(11.12) = 0.62, *p* = 0.810, $\eta_{p}^{2}$ = 0.051, and the between-subjects factor of experimental group had no significant effect, *F*(4) = 1.55, *p* = 0.203.

### Sense of Control Intervention on Task Performance

Task performance analyses compared the second and last blocks (blocks when training was applied). Over the anagram task, a decrease in accuracy was seen for Group 4, *T* = 2.5, *p* = 0.047, *r* = -0.423, and no experimental group showed a significant change in speed.

Over the CIC task, a decrease in accuracy was seen only for the groups not receiving sense of control training: Group 2 (*Mdn* 5 to 4.5; *T* = 0, *p* = 0.041), Group 4 (*Mdn* 5 to 4, *T* = 0, *p* = 0.026), and Group 5 (*Mdn* 5 to 4.5; *T* = 0, *p* = 0.038). Reaction times decreased for Group 3, *T* = 52, *p* = 0.013, *r* = 0.558, and Group 4 showed a non-significant trend toward decreasing speed, *T* = 52, *p* = 0.091.

In the ABM task, there was no significant effect of time on accuracy, *F*(1) = 0.49, *p* = 0.489, and no significant time-group interaction, *F*(1) = 1.15, *p* = 0.347. Reaction time data was only examined with respect to probe conditions in the attention bias analyses.

### Sense of Control Intervention on Alcohol Craving

Binge drinkers reported higher past-week craving as measured on the PACS (*Mdn* = 7) than NBDs (*Mdn* = 2), *U* = 37, *p* < 0.001, *r* = -0.561, but the two groups did not differ on craving at the start of the experiment (TLC; *Mdn* = 0 for both drinking groups), (*U* = 177.5, *p* = 0.285, *r* = -0.114).

Time-locked craving ratings changed over time for Group 2, $\chi_{F}^{2}$ = 6.50, *p* = 0.020. The point of difference was not detectable with pairwise comparisons, but a Wilcoxon’s signed rank test identified an increase from before the attention intervention (*Mdn* = 0, interquartile range = 1) to after (*Mdn* = 1, interquartile range = 3), *T* = 15, *p* = 0.020, *r* = 0.461. Group 4 showed a similar increase, $\chi_{F}^{2}$ = 4.63, *p* = 0.049, from before ABM (*Mdn* = 0) to after ABM (*Mdn* = 2). No other experimental group reported a change in craving.

### Effects of Sense of Control Intervention on Alcohol Consumption

The 2 × 5 (Beverage × Experimental group) ANOVA identified a significant main effect of beverage, *F*(1) = 4.72, *p* = 0.019, *d* = 0.79. Participants overall consumed more orange juice (*M* = 65.66 g, *SD* = 54.05) than beer (*M* = 53.29 g, *SD* = 60.69). There was a significant Beverage × Group interaction, *F*(4) = 2.91, *p* = 0.019, $\eta_{p}^{2}$ = 0.280, where experimental groups drank the same amount of orange juice, but differed in beer consumption: Groups 4 and 5 drank significantly more beer (respectively, *M* = 81.13 g, *SD* = 79.22, and *M* = 91.50 g, *SD* = 85.52) than Group 1 (*M* = 33.25 g, *SD* = 22.83; *p* < 0.001).

## Discussion

Binge drinking, like other patterns of hazardous drinking, has been associated with attention bias for alcohol, which has implications for continued excessive use and craving. The current study was designed to investigate alcohol attention bias in BDs, its possible reduction, and whether increasing sense of control in addition to ABM would improve outcomes for alcohol attention bias, craving and consumption. Overall, the findings showed BDs had higher alcohol attention bias than NBDs, that an ABM intervention alone was not able to decrease this trend, but that a combined intervention involving sense of control training decreased alcohol consumption.

Our finding of a higher alcohol attention bias in BDs is consistent with previous findings in comparable groups (Townshend and Duka, 2001; Field et al., 2004; Field and Eastwood, 2005) and elsewhere student BDs have shown attention bias deficits (Petit et al., 2012, 2014). This was not supported by ERP measures in the present study. The current study used a long stimulus presentation time of 500 ms, which reflects difficulty disengaging from cues (Field and Cox, 2008). Elsewhere, ERP evidence has identified heavy drinkers’ attention bias using this long stimulus presentation (e.g., Townshend and Duka, 2001; Field et al., 2004; Field and Eastwood, 2005); whether BDs show speeded cue detection with shorter presentation times remains to be explored.

ABM did not reduce BDs’ attention bias, likely because of the relatively low baseline scores. Use of an untrained, rather than active (e.g., Field and Eastwood, 2005), control group also attenuates any difference between groups, as in this case, but is more clinically relevant. Additionally, it may take repeated sessions to reveal a training effect, although it is noted that Schoenmakers et al. (2007) showed single-session effects in heavy drinkers using similar parameters (e.g., picture stimuli, top-bottom arrangement). Finally, the choice of probes (↑ or →) may have affected results by directing participants’ attention to the cue on the right of the arrow probe, instead of to the cue which was replaced by the probe. More neutral cues should be used in replication (e.g., ↕ or ↔).

Attention bias scores for all participants decreased over time, regardless of group or training, suggesting habituation on cue exposure, perhaps because the difference between BDs and NBDs at baseline was not marked. Neurophysiological measures added some nuance to this finding: ERPs showed all BDs, regardless of training group, shifted over time from an alcohol-focus to a neutral-focus, and that NBDs did the reverse. This was an unexpected finding that suggests attentional habituation with cue exposure for the former and a sensitisation for the latter. Alcohol cues were more resistant to habituation, shown in the time-probe interaction for participants’ frontal N1 amplitudes, where showed only amplitudes to neutral cues decreased over time. This resistance may represent a point of difference between the neural mechanisms governing appetitive biases versus threat biases, and a challenge to appetitive ABM.

Regarding the sense of control intervention, the current study did not replicate Shamloo and Cox (2014), who reported that the sense of control intervention can increase tasks-specific sense of control; here, task-specific sense of control decreased for all participants, regardless of training. Overall accuracy scores suggest the tasks, particularly the anagrams, may have been too challenging to impart feelings of success critical to the sense of control intervention (Shamloo and Cox, 2014). Provided tasks are at an appropriate difficulty level, the sense of control training could improve task accuracy. Sense of control training did not affect reaction times, possibly because greater sense of control could manifest as perseverance rather than speed (Shamloo and Cox, 2014), and so accuracy can be a more useful measure. Only untrained groups became less accurate in the CIC tasks, suggestive training was protective. Additionally, untrained participants (Groups 1 and 3) showed an increase in craving over the ABM task, while trained participants showed no change. Again, sense of control training could be considered protective. Without the support of the sense of control intervention, the cognitive challenge of the difficult tasks may have triggered automatic action schema that promote craving (Tiffany, 1990) in the untrained groups, especially when craving was further challenged by exposure to alcohol cues in the following ABM task.

Effort was made to replicate the original measures, and validate summary scales with a pilot group, but slight differences between the studies may exist. For example, the authors report using the SCI’s “Overall” subscale, but their description better fits the “Domain-specific” subscale.

The combined intervention did not reduce attention bias, but did influence alcohol consumption in the voluntary taste-test. Participants receiving both interventions, but not participants receiving one intervention, drank less alcohol than those receiving none. This could be the work of a unique combination of protection against craving, from the sense of control intervention, and reduced attention bias, from cue-exposure and habituation if not ABM. NBDs’ high alcohol consumption was unexpected but could be explained by personality characteristics: for example, NBDs tend to be more conscientious (Ichiyama and Kruse, 1998) and, being fastidious test-takers, may have consumed more of the beverage with which they are less familiar.

Few BDs committed to the study despite many eligible BDs expressing interest at the screening stage. In this regard, we cannot exclude the possibility that the study was not sufficiently powered to detect differences between groups. Similarly, despite varied recruitment efforts, young people and students were overrepresented in the final sample, but it is known binge drinking is not just a youth issue and BDs are most likely to be full-time wage or salary earners (McMillen et al., 2004). To distinguish the effects of global alcohol intake from the specific pattern of consumption, the groups of Maurage et al. (2012) could be used: control non-drinkers, daily drinkers, low and high BDs. Beyond addressing these sample issues, an interesting extension for future study would be to apply the sense of control intervention elements directly to the ABM task as follows: choice (of personalized stimuli set), information enhancement (on-screen tips or reminders of the task’s skills-based nature), goal-setting (accuracy goals) and reinforcement. This could be achieved in a task *gamification*, which has been successful in ABM for anxiety (Dennis and O’Toole, 2014). It would also further standardize the ABM task. It would be interesting to see whether the protective effect against craving of the sense of control manipulation remains when the intervention does not precede cue-exposure. Finally, targeting automatic *approach bias* to appetitive drug cues (rather than just the attention bias, although they are related) has shown promise (Eberl et al., 2013). Training to discourage cue approach may have more dramatic effects than the subtle priming of ABM.

In conclusion, BDs, defined with a well cited measurement, exhibited increased baseline alcohol attention bias concomitant with increased consumption in comparison to NBDs. Nonetheless, BDs may be a more heterogeneous group than academic, policy, or popular conceptions portray, and researchers should be wary of the catch-all term, defined rather confusedly in the literature as is (Herring et al., 2008). Future research is tasked with considering a diversity of BDs, without understating the risk of harm. Furthermore, the study offers mixed support for the two brief interventions. While the sense of control intervention did not increase personal or task-specific sense of control, it holds potential for protecting BDs from craving increases otherwise seen in the untrained when exposed to alcohol cues. The ABM intervention did not decrease attention bias, but when combined with sense of control training, resulted in lower alcohol consumption than no training. These findings should be interpreted with some caution, however, due to the relatively small sample size and the lack of strong behavioral effects when these interventions were administered separately.

## Author Contributions

JL collected the data. All authors equally contributed to the study design, data analysis, and writing the manuscript.

## Conflict of Interest Statement

The authors declare that the research was conducted in the absence of any commercial or financial relationships that could be construed as a potential conflict of interest.
